# Scanning of antennae and maxillary palps of anthropophilic *Aedes aegypti* and ornithophilic *Culex pipiens* as potential arbovirus vectors

**DOI:** 10.14202/vetworld.2024.2248-2252

**Published:** 2024-10-07

**Authors:** Faten Abouelmagd, Mohamed Elmutasim Elsheikh, Elshiekh Khidir, Mohammed Radwan, Karim Mohamed Rashad, Manal El Said

**Affiliations:** 1 Department of Medical Parasitology, Faculty of Medicine, Sohag University, Sohag, 82524, Egypt; 2 Department of Microbiology, General Medicine Practice Program, Batterjee Medical College, Jeddah, 21442, Saudi Arabia; 3 Department of Laboratory Medicine, Faculty of Applied Medical Sciences, Umm Al-Qura University, Al-Abdeyah, Makkah, 24381, Saudi Arabia; 4 General Medicine Practice Program, Batterjee Medical College, Jeddah, 21442, Saudi Arabia; 5 Kasr Alainy, Faculty of Medicine, Cairo University, Giza, 11562, Egypt; 6 Department of Microbiology and Infection Prevention and Control Unit, Theodor Bilharz Research Institute, Giza, 12411, Egypt

**Keywords:** *Aedes aegypti*, *Culex pipiens*, Scanning electron microscopy, Sensilla

## Abstract

**Background and Aim:**

Efficient mosquito vectors are required to persist and propagate arthropod-borne diseases that seriously affect impoverished populations worldwide. Mosquito sensilla plays a crucial role in host-seeking and disease transmission to humans. This study aimed to distinguish between the several types of sensilla found on the antennae and maxillary palps of *Culex pipiens* and *Aedes aegypti*, matching this diversity with host preference and disease transmission.

**Methods:**

Overall, 1300 mosquitoes were collected and examined using dissection and light microscopy. Scanning electron microscopy was used to identify and describe the diverse types of sensilla found on the antennae and maxillary palps of *C. pipiens* and *A. aegypti*.

**Results:**

In total, 900 *C. pipiens* and 400 *A. aegypti* mosquitoes were identified. The antennae and maxillary palps of *C. pipiens* and *A. aegypti* carry both sensilla trichoidea and sensilla chaetica. The *C. pipiens* antenna has long and short grooved peg sensilla, whereas *A. aegypti* lacks long pegs and expresses only occasional short pegs. The maxillary palps express Capitate pegs in both mosquito species and exclusively show sensilla campaniform in *A. aegypti*.

**Conclusion:**

The lack of long-grooved pegs and the presence of few short pegs, along with campaniform sensilla, limit the host range of *A. aegypti* and reduce its susceptibility to many infections, unlike *C. pipiens*.

## Introduction

The diversity of arthropod-borne illnesses that affect human health directly or through the affection of domestic animals and livestock is transmitted through mosquitoes, which serve as biological vectors for these diseases [1]. The antennae, which carry olfactory sensory neurons, are the primary olfactory organs in mosquitoes [2]. Signaling received through the sensilla controls the female mosquito cycle and manages their life behavior by reacting to various olfactory stimuli, such as the location of oviposition, nectar, mating, and hosts [3]. When mosquitoes seek hosts, their sensilla and sensory mechanisms facilitate host identification and the spread of a variety of infections to humans [4]. Host-seeking tends to be an odor-guided behavior; however, mosquitoes also use multimodal integration of sensory inputs to identify their blood hosts [5].

Mosquito host-seeking is detected by neurons in the sensory appendages. The main chemosensory appendages of mosquitoes involved in host search include the antennae and maxillary palps. Some mosquitoes may have additional olfactory neurons that express a receptor that responds to human-derived odors, such as decanal or decanoic acid, and thus confer higher sensitivity to human hosts. Conversely, additional neurons may express an olfactory receptor that is more sensitive to animal odors, making the individual more amenable to alternating blood hosts. The type of sensilla and the number of neurons in each sensilla play key roles in host detection [6].

Mosquitoes exhibit various innate feeding preferences in natural habitats. While some species, including some anthropophilic species, primarily bite mammals (mammophilic), others prefer to bite birds (ornithophilic species) [7]. *Culex pipiens* and *Aedes aegypti* (Diptera: Culicidae) are arbovirus vectors. *C. pipiens* displays a mammalian host-feeding preference, whereas *A. aegypti* mainly bites human beings [8–10].

This study aimed to compare the sensilla types on the antennae and maxillary palps of *C. pipiens* and *A. egypti*, which were obtained from Jeddah City, Saudi Arabia, and to examine sensilla differences in correlation to host selection and disease transmission.

## Materials and Methods

### Ethical approval

This study was approved by the research unit of Batterjee Medical College (Approval No.: RES-2023-0040).

### Study period and location

Adult mosquitoes were collected from Jeddah city in Saudi Arabia between September 2022 and March 2023. Mosquito identification was conducted at Batterjee Medical college.

### Mosquito collection and identification

Adult mosquitoes (males and females) were collected from Jeddah City, Saudi Arabia, using mechanical aspirators, spray-sheet collection, and box traps. In total, 900 *C. pipiens* and 400 *A. aegypti* mosquitoes were collected. The mosquitoes were directly examined using dissection and light microscopy. Taxonomic keys were used for the taxonomic identification of adult mosquitoes [11–14]. The average number and percentage of each mosquito species and the average and percentage of females in each species were determined.

### Electron microscopic examination of the mosquito antenna and maxillary glands

Electron microscopic examination of the antennae and maxillary palps of the identified adult female mosquitoes was performed by washing the mosquito heads using an ultrasonic cleaner for 30 s. Then, they were dehydrated using an ascending series of ethanol (70%, 80%, 90%, and 100%) for 25 min per treatment and then air-dried. The specimens were fixed onto scanning electron microscopy (SEM) stubs and coated with gold. Examinations of the mosquito antennae and maxillary palps by SEM were performed using a Hitachi S-3400N (Hitachi, Tokyo, Japan) at an accelerating voltage of 15 kV [15].

## Results

**Figure-1:**
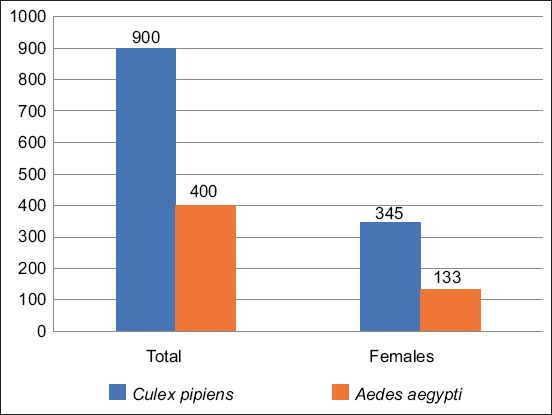
Number of female mosquitoes relative to the total number of mosquitoes in the study.

*C. pipiens* (900; 69.2%) and *A. aegypti* (400; 30.7%) were identified using dissection and light microscopy, respectively. Female *C. pipiens* (345/900; 38.3%) and *A. aegypti* (133/400; 33.3%) are shown in (Figure-1).

**Figure-2:**
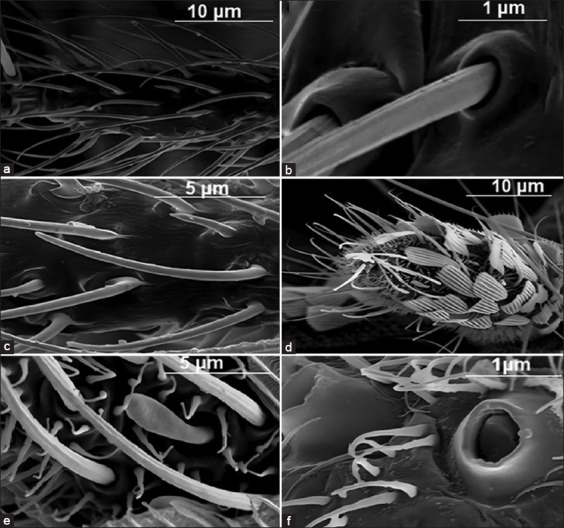
(a) Antennal flagellomere with short and long sensilla trichoidea and sensilla chaetica at the flagellomere base, (b) Sensilla chaetica grooved emerging from deep sockets, (c) Short grooved pegs, (d) Maxillary palp with sensilla trichoidea and sensilla chaetica along with abundant scales and microtrichia, (e) Capitate peg arising from a circular depression with broad tip, and (f) Sensilla campaniformia with a hinged band of elevated cuticle around.

The antennae of *C. pipiens* and *A. aegypti* are composed of three components: scape, pedicel, and flagellum, with 13 flagellomeres covered by cuticle-like projections, scales, and microtrichia. The two adult mosquito species examined carried both sensilla trichoidea and sensilla chaetica, which had similar morphologies and distributions. Sensilla trichoidea was the most common mosquito species. Sensilla trichoidea are hair-like structures found along flagellomeres. They are found in approximately equal numbers and distributions. These came from shallow sockets. They range in length of 10–55 μm and are categorized into the following types: Blunt or pointed and either short or long (Figure-2a). Sensilla chaetica are long, stiff, ridged, hair-like structures that appear radially dispersed along the flagellomere and are set into whorls at their base. These sensilla are the longest, ranging in length from 300 to 315 μm. They have thick walls that are outwardly grooved and emerge from deep sockets with sharp pointed ends (Figure-2b). The grooved pegs were thick-walled and extensively grooved. They range in length from 5 μm to 10 μm (Figure-2c). Grooved pegs have two subtypes: pointed long pegs and blunt short pegs. *C. pipiens* antenna shows both types of grooved peg sensilla, whereas *A. aegypti* lacks long pegs and expresses only occasional short pegs.

The maxillary palps of *C. pipiens* and *A. aegypti* had the same sensilla trichoidea and sensilla chaetica, with abundant scales and microtrichia (Figure-2d). In addition, the Capitate pegs are presented on both mosquito species, which are 12.5 ± 1 μm in length and have a lyre-shape, broad tip ending, and arise from a circular depression (Figure-2e). *A. aegypti* mosquito maxillary palps have sensilla campaniformia that are dome-shaped and 5.4 μm in diameter, with a hinged band of elevated cuticle around it and situated on the distal end of the maxillary palps (Figure-2f). Sensilla campaniformia were absent from the maxillary mosquitoes of *C. pipiens*.

## Discussion

Mosquito sensilla are found in specific places on the body, particularly in the antennae, maxillary palps, proboscis, tarsi, and tergum. The sensilla have various shapes. Each stimulus was designed to respond to a specific stimulus. The presence or absence of certain types of sensilla may affect mosquito host preference and disease transmission [16]. Based on the results obtained, the difference in the types of sensilla between *C. pipiens* and *A. aegypti* affects their host preferences and disease transmission. The presence of sensilla campaniformia in *A. aegypti* enhances its host selection compared to *C. pipiens*.

The ability of female mosquitoes to transmit diseases depends on their behavioral responses to the host. Mosquitoes can distinguish and seek hosts by sensing CO_2_, heat, body odor, visual signs, and water vapor. Sensilla campaniformia, grooved pegs, and sensilla trichoidea are sensory sensilla that respond to these stimuli. Sensilla chaetica and grooved peg sensilla are tactile receptors [6].

Mosquito blood feeding is influenced by odors and directly affects disease transmission [17]. Many human mosquito-borne diseases are zoonoses with amplification cycles involving species other than humans. *C. pipiens* is among the most abundant bird feeders, whereas *A. aegypti* is mostly anthropophilic and occasionally engorged with avian blood [18], which is transmitted by anthropophilic *A. aegypti*, which propagates over large distances by exploiting human movement to infect non-cautious countries.

The antennae and maxillary palps play key roles in host detection and other sensory-mediated behaviors. In this study, several types of sensilla found on the antennae and maxillary palps of *C. pipiens* and *A. aegypti* were identified, and their diversity was correlated with host preference and disease transmission.

The current study showed that both *C. pipiens* and *A. aegypti* expressed sensilla trichoidea and sensilla chaetica on their antennae and maxillary palps, with similar distribution patterns. Both forms of grooved peg sensilla are presented on the antennae of *C. pipiens*, whereas *A. aegypti* lacks long pegs and only occasionally displays small pegs. However, a previous study by Mciver [19] identified two subtypes of grooved peg sensilla in some *Aedes* and *Culex* species. A potential change in the manner in which odor molecules are perceived is indicated by the notable length difference between the two types of grooved peg sensilla [20].

In this study, maxillary palp capitate pegs were observed in both *C. pipiens* and *A. aegypti*, whereas *A. aegypti* was the only species found in sensilla campaniformia. In another report, *A. aegypti* exhibited a pair of sensilla campaniformia at the tarsal margin [21, 22].

In contrast to *C. pipiens*, which transmits a broad range of diseases, *A. aegypti* may be more refractory to certain diseases owing to its lack of long grooved pegs, scarcity of short pegs, and presence of campaniform sensilla [22]. In Saudi Arabia, many studies have discussed the distribution of arboviruses transmitted by *C. pipiens* and *A. aegypti* and how their host preferences among birds, mammals, and humans may affect disease spread and reporting, especially during epidemics and interepidemic phases.

*C. pipiens* is one of the most prevalent and widely dispersed mosquito species in Saudi Arabia. It is primarily responsible for the transmission of arboviruses, including the West Nile virus (WNV), which is most common in the Middle East. The primary vector of WNV is *C. pipiens*, which feeds primarily on non-human hosts during disease transmission [23]. *C. pipiens* is frequently described as ornithophilic. Therefore, bird migration and viral dynamics that depend on mosquito population activity are the main factors influencing WNV transmission [18].

WNV is maintained in a natural zoonotic transmission cycle between birds and mosquitoes; however, it can be transmitted through mosquitoes to mammals, including humans. However, humans act as dead-end hosts because the viremia in humans caused by avian arboviruses is too low to infect biting vectors. As a result, mammals and humans are generally viewed as dead-end hosts. Therefore, >80% of WNV infections in humans are asymptomatic, and many cases go unreported or undiagnosed [24]. The widespread distribution of the competent vector, *C. pipiens*, and migratory birds can explain WNV infections in certain areas [25].

In a study conducted in Saudi Arabia, WNV circulation in horses was evident in the Eastern and Central Regions of the country [26]. Another study conducted by Alkharsah and Al-Afaleq [27] in Saudi Arabia provided the first evidence of anti-WNV antibody detection in humans and pigeons. Evidence indicates that WNV is widespread in Saudi Arabia [27].

The Indian and African oceans were considered the only oceans where Rift Valley fever virus (RVFV) epidemics might have occurred. Arab countries, such as Egypt, Sudan, and Somalia, have already reported RVFV. The first RVFV outbreak was reported in Saudi Arabia in 2001, with 884 hospitalized patients and 124 fatalities [28]. An unknown hemorrhagic fever in humans was reported by the Saudi Arabian Ministry of Health. However, approximately 50% of infected individuals exhibit clinical symptoms, whereas others may display flu-like symptoms [29].

In Saudi Arabia, *C. pipiens* is a potential RVFV vector. Mosquitoes transmit RVFV to animals, primarily cattle, sheep, goats, and camels, whereas birds are infection-resistant [30]. *Aedes* mosquitoes are the primary vector of RVFV, but *C. pipiens*, which is considered a secondary vector of RVFV, has the best ability to transmit the virus horizontally from highly viremic animals to humans because of its wider host range compared with *A. aegypti* [29].

Thousands of workers reside in Saudi Arabia each year, in addition to millions of pilgrims who perform Hajj and Umrah. Consequently, the likelihood of imported mosquito-borne infectious diseases has increased. Furthermore, it is highly likely that such diseases will become endemic once they are introduced into the region because of the abundance of vectors [23].

## Conclusion

Sensilla plays a distinct role in host detection and disease transmission. The presence or absence of certain types of sensilla may determine mosquito host preference and affect the mosquito-borne disease cycle. *A. aegypti*’s host range is restricted by the lack of long-grooved pegs, the presence of rare short pegs, and the presence of campaniform sensilla, which reduce the susceptibility of mosquitoes to numerous diseases. This is in contrast to *C. pipiens*, which is a more powerful disease transmitter. Mosquito collection is challenging because the distribution and abundance of mosquito species are influenced by many factors, such as host availability, human activities, and climatic conditions. Further studies with more significant numbers of mosquito species are required to better identify the wide-ranging sensilla in relation to diverse mosquito-borne infections.

## Data Availability

The supplementary data can be available from the corresponding author on a reasonable request.

## Authors’ Contributions

ME and FA: Study conception and design. FA, ME, MEE, EK, MR, and KMR: Sample collection, material preparation, result extraction, and data analysis. FA and ME: Drafted the manuscript. All authors have read, reviewed, and approved the final manuscript.
